# Public Knowledge, Attitudes, and Experience Regarding the Use of Antibiotics in Italy

**DOI:** 10.1371/journal.pone.0084177

**Published:** 2013-12-23

**Authors:** Francesco Napolitano, Maria Teresa Izzo, Gabriella Di Giuseppe, Italo F. Angelillo

**Affiliations:** Department of Experimental Medicine, Second University of Naples, Naples, Italy; Institut National de la Recherche Agronomique, France

## Abstract

**Background:**

The objectives of the study were to investigate the level of knowledge, attitudes, and behaviors regarding antibiotics of the general population in Italy, and to assess the correlates of these outcomes of interest.

**Methods:**

A cross-sectional survey was conducted on a random sample of 630 parents of students attending nine randomly selected public primary and secondary schools. A self-administered questionnaire included questions on demographic characteristics, knowledge about antibiotic use and resistance, attitudes and behaviors towards antibiotic use, and sources of information.

**Results:**

A total of 419 parents participated. Only 9.8% knew the definition of antibiotic resistance and 21.2% knew when it was appropriate to use antibiotics. Respondents with higher education, employed, with a family member working in the health care sector, and with no need for additional information on antibiotics were more likely to know the definition of antibiotic resistance. One third (32.7%) self-classified them as users of self-medication with antibiotics and those with a lower self-rated health status, who did not use the physician as source of information on antibiotics, and who have attended a physician in the last year were more likely to use self-medication. One-fourth (22.7%) of those who had never been self-medicated would be willing to take an antibiotic without a prescription of a physician. Respondents were more likely to be willing to take antibiotics without a prescription if they were under 40 years of age, if they had a lower self-rated health status, if they did not know that antibiotics are not indicated for treating flu and sore throat, and if they knew that antibiotics are not indicated for treating colds.

**Conclusions:**

The survey has generated information about knowledge, attitudes, and behaviors regarding antibiotics in the general population and effective public education initiative should provide practical and appropriate means to change their behavior.

## Introduction

The overuse and misuse of antibiotics may generate several problems, including the development of bacterial resistance [1]–[3], the rising costs of health-care services [4], [5], and the development of side effects [5], [6]. Studies have consistently documented all over the world that the inappropriate and excessive use of antibiotics are the predominant factors that causes the emergence and selection of resistant bacteria with the result of antibiotic resistance that represents one of the most important worldwide issue for global public health and for patient-safety [1]–[3], [6]–[11]. The inappropriate and excessive antibiotic utilization in the community, in primary care, and in hospitals may result from a complex interaction between several factors such as, for example, the practices of physicians, the patients' attitudes, beliefs, knowledge of antibiotic use, the self-medication, the patients' perceptions regarding patient-physician interaction, the patients' expectations, and the patients' experience with antibiotics [12]–[15]. Therefore, controlling the antibiotic use requires a multifaceted approach with knowledgeable and engaged health-care professionals, pharmacists, health authorities, and consumers.

The general population can play an important role in reducing the inappropriate and excessive utilization of antibiotic and it is necessary to understand their antibiotic use knowledge, attitudes, and behaviors and if any educational needs exist. Despite a number of research reports in the literature investigated around the world the antibiotic use knowledge, attitudes, and behaviors of the general public [2], [10], [16]–[19], of secondary school teachers and University members [20], of students [21], [22], of primary care center attendants [23], and of parents [24], [25], to our knowledge there are no studies carried out in Italy. As a result, it is important to measure this phenomenon. Therefore, the objective of the current study was firstly to investigate the level of knowledge, attitudes, and behaviors regarding antibiotics of the general population in Italy. The secondary aim of this study was to identify the factors that were linked to the main outcomes of interest.

## Materials and Methods

This was a cross-sectional epidemiologic survey conducted in December 2011 designed to sample randomly 630 parents of students between the age of 5–18 years old attending nine randomly selected public primary and secondary schools in the geographic area of Caserta and Naples, Italy. In order to interview the parents, the study proposal was discussed with the directors of the selected schools and approval was obtained.

Students were asked to take home a sealed envelope, including an introductory letter, an informed consent form, a two-page questionnaire which was designed to collect self-report information, and a self-addressed envelope for returning the survey, which were collected by contact persons at the schools and delivered to the organizers of the survey. The letter included a brief description of the study objectives and its importance, inviting only one self-identified parent from each family to complete the questionnaire. Participants were assured that participation in the survey was voluntary, about confidentiality of the information to be provided, and security and privacy of responses. The confidentiality of the information was maintained by excluding personal identifiers, and all the data collected were processed and analyzed anonymously. All participants provided written informed consent at the beginning of the survey prior to answering any question by reading the consent form. The interviewees did not receive any financial or other compensation for participation in the study.

The number of parents to be randomly sampled in this study was determined based on the assumptions that 30% of the population know the appropriate use of antibiotics, using a confidence interval of 95%, a margin of error of 5%, and an expected response rate of 50%. It was estimated that it would need to enroll a total number of 485 parents for the study.

The questions in the questionnaire were grouped broadly into the following five categories: (a) socio-demographic characteristics of the participants (gender, age, marital status, educational level, employment status, number and age of sons, number of cohabiting, partner's employment status, health-care worker in the family); (b) knowledge about antibiotic use and antibiotic resistance; (c) attitudes toward self-medication with antibiotics; (d) self-reported practices related to antibiotics use; and (e) sources and needs of information regarding antibiotics use. The questionnaire consisted of closed and open-ended questions, regarding demographic characteristics, knowledge about antibiotics use and resistance, attitudes and behaviors towards antibiotics use, and sources of information. The respondent's knowledge was tested with tree questions relating antibiotics use, with “yes, no, do not know” responses, and one about the definition of antibiotic resistance in open format. Their practices about antibiotics use were measured with four questions which required a nominal or categorical (yes or no) responses. One question about compliance with the antibiotic's prescription was measured on a five-point Likert scale ranging from “never” to “every time”. Respondents were asked whether they had ever taken an antibiotic without a prescription. They were classified as users of self-medication if they reported that they had ever taken any antibiotic without a prescription. Finally, in the question regarding the information sources, respondents were able to indicate more than one source.

To verify the quality of the questionnaire, the clarity of the questions, and the length of the latter, a pilot study was conducted on a population of 20 volunteers and subsequently reconstructed some of the questions. Taking into account all ethical requirements, the project was institutionally approved by the Ethical Committee of the Second University of Naples.

### Statistical analysis

A multivariate analysis was performed by binary logistic regression test. Considering all of the participants, factors affecting the three following dependent variables were assessed by multiple logistic regression analysis: knowledge of the definition of antibiotic resistance (non correct response  = 0, correct response  = 1) (Model 1), self-medication with antibiotics (no  = 0, yes  = 1) (Model 2), and willingness to take antibiotics without a prescription of a physician (no  = 0, yes  = 1) (Model 3). In all models the following respondent characteristics were included: gender (male  = 0, female  = 1), age (three categories: <40 years  = 1, 40–45 years  = 2, >45 years  = 3), educational level (three categories: middle school or lower  = 1, high school  = 2, baccalaureate degree/graduate degree  = 3), employment status (unemployed  = 0, employed  = 1), a health-care worker in the family (no  = 0, yes  = 1), having attended a physician at least once in the last year (no  = 0, yes  = 1), physicians as source of information on antibiotics (no  = 0, yes  = 1), and need for additional information on antibiotics (no  = 0, yes  = 1). The following variables were also included in Models 2 and 3: self-rated health status (continuous), knowledge that antibiotics are not indicated for treating fever (no  = 0, yes  = 1), knowledge that antibiotics are not indicated for treating sore throat (no  = 0, yes  = 1), knowledge that antibiotics are not indicated for treating flu (no  = 0, yes  = 1), and knowledge that antibiotics are not indicated for treating cold (no  = 0, yes  = 1). Stepwise technique was utilized. The significance level for variables entering in the logistic regression models was set at 0.2 and for removing from the model at 0.4. Odds ratio (ORs) with 95% confidence intervals (CIs) were calculated. A two-sided *p*-values of 0.05 or less indicated statistical significance. Using the statistical package Stata (version 10.1) [26], descriptive and inferential statistical analyses were performed to examine and compare the responses obtained.

## Results

### Participants' characteristics

In total, 630 questionnaires were distributed and 419 parents provided informed consent to participate and returned a completed questionnaire amenable to analysis, representing a response rate of 66.5%. The characteristics of the respondents are summarized in Table 1. Two-thirds of the sample were females (71.1%), the ages ranged from 28 to 60 with an average of 43.1, two-thirds had completed at least the high school (65.4%), and more than half were employed (54.2%).

**Table 1:** Principal characteristics of the study population.

	Total	
	n	%
*Gender*		
Male	121	28.9
Female	298	71.1
*Age* (*years*)	43.1±6.1(28–60)*	
<40	118	28.2
40–45	150	35.8
>45	151	36
*Marital status*		
Married	389	92.8
Other	30	7.2
*Educational level*		
Middle school or lower	145	34.6
High school	225	53.7
Baccalaureate degree/Graduate degree	49	11.7
*Employment status*		
Unemployed	189	45.8
Employed	224	54.2
*Number of cohabiting*		
≤3	294	70.4
>3	125	29.6
*Number of sons*		
1	54	12.9
>1	365	87.1
*A health-care worker in the family*		
Yes	107	25.5
No	312	74.5
*Self-rated health status*	7.6±1.6(1–10)*	
*Having attended a physician at least once in the last year*		
No	76	18.1
Yes	343	81.9

*Mean ± standard deviation (range).

Numbers for employment status do not add up to the total number of the study population due to missing values.

### Participants' knowledge

When assessing the level of knowledge amongst the participants, only 9.8% correctly knew the definition of antibiotic resistance, 21.2% knew when it is appropriate the antibiotics use, 50% and 83% respectively knew that the antibiotics lose their effectiveness if the treatment is interrupted and if the prescription is not respected. Further, most of the participants are able to correctly answer that antibiotics must not be used if they have a cold (80.7%), on the other hand lower values have been observed regarding the fact that antibiotics must not be used for sore throat (55.1%), flu (50.1%), and fever (42.7%). Table 2 present the factors that contributed to the different outcomes of interest according to the multivariate logistic regression analyses. The results revealed that four variables were significantly and independently positively associated with the knowledge of the definition of antibiotic resistance (Model 1). Respondents who were employed and who have a family member working in the health-care sector were respectively 3.1 (95% CI = 1.2–7.6) and 2.2 (95% CI = 1.1–4.5) times as likely to know the definition compared to unemployed and to who have not a health-care worker in the family. Those who have no need for additional information on antibiotics (OR = 0.46; 95% CI = 0.23–0.92) were more likely to answer the item correctly. Last, when the baccalaureate degree/graduate degree was chosen as reference category, the odds of knowledge of the definition of antibiotic resistance was significantly lower among those with a middle school or lower educational level compared with those with a higher level (OR = 0.1; 95% CI = 0.03–0.4).

**Table 2:** Regression models for potential determinants of the different outcomes of interest.

Variable				
**Model 1. Knowledge of the definition of antibiotic resistance (** ***n*** ** = 419)**	**OR**	**SE**	**95% CIs**	***p*** ** value**
Log likelihood = −110.75, χ^2^ = 46.9 (7 df), *p*<0.0001				
Educational level				
Baccalaureate degree/Graduate degree	1*			
Middle school or lower	0.1	0.08	0.03–0.4	0.002
High school	0.6	0.2	0.26–1.31	0.193
Employment status	3.1	1.4	1.2–7.6	0.014
A health-care worker in the family	2.2	0.8	1.1–4.5	0.024
Need for additional information on antibiotics	0.46	0.16	0.23–0.92	0.028
Age (years)				
>45	1*			
<40	0.46	0.24	0.17–1.26	0.134
Physicians as source of information on antibiotics	1.6	0.7	0.65–3.96	0.309
**Model 2. Self-medication with antibiotics (** ***n*** ** = 419)**	**OR**	**SE**	**95% CIs**	***p*** ** value**
Log likelihood = −253.24, χ^2^ = 23.14 (6 df), *p* = 0.0007				
Physicians as source of information on antibiotics	0.48	0.11	0.3–0.77	0.003
Self-rated health status	0.85	0.06	0.74–0.97	0.017
Having attended a physician at least once in the last year	1.88	0.58	1.03–3.43	0.04
Need for additional information on antibiotics	0.77	0.17	0.49–1.19	0.244
Knowledge that antibiotics are not indicated for treating cold	1.32	0.37	0.75–2.3	0.328
Age (years)				
>45	1*			
<40	1.23	0.3	0.77–1.96	0.376
**Model 3. Willingness to take antibiotics without a prescription of a physician (** ***n*** ** = 282)**	**OR**	**SE**	**95% CIs**	***p*** ** value**
Log likelihood = −137.49, χ^2^ = 27.1 (9 df), *p* = 0.0014				
Age (years)				
>45	1*			
<40	2.34	0.77	1.22–4.5	0.01
Knowledge that antibiotics are not indicated for treating flu	0.42	0.14	0.22–0.82	0.011
Self-rated health status	0.79	0.07	0.65–0.95	0.012
Knowledge that antibiotics are not indicated for treating sore throat	0.51	0.16	0.27–0.95	0.034
Knowledge that antibiotics are not indicated for treating cold	2.49	1.1	1.04–5.95	0.04
Educational level				
Baccalaureate degree/Graduate degree	1*			
Middle school or lower	0.5	0.25	0.18–1.36	0.177
High school	0.63	0.3	0.24–1.61	0.332
Physicians as source of information on antibiotics	0.63	0.22	0.31–1.27	0.198
Gender	0.73	0.25	0.38–1.43	0.362

*Reference category.

### Participants' behavior

With regard to the behaviors, 87.9% of the respondents had attended a physician at least once in the twelve months preceding the survey, and more than three-quarters (79.3%) of them have received a prescription of antibiotics and approximately two-third (63.6%) have ever respected the prescription. Regarding the use of antibiotics, one-third (32.7%) of the respondents self-classified them as users of self-medication since they had taken an antibiotic without the prescription of a physician. When the impact of the many potential predictor variables of the use of self-medication with antibiotics was investigated using multivariable logistic regression, three variables have a significant influence on this outcome. Respondents with a lower self-rated health status (OR = 0.85; 95% CI = 0.74–0.97), those who did not use the physician as source of information on antibiotics (OR = 0.48; 95% CI = 0.3–0.77), and those who have attended a physician in the last year (OR = 1.88; 95% CI = 1.03–3.43) were more likely to use self-medication with antibiotics (Model 2 in Table 2). The self-medication with antibiotics was mainly to treat throat (29.1%), fever (21.2%), teeth symptom (20.4%), and common cold (17.5%). The reasons reported by the participants for using no prescribed antibiotics were: the physician was not available (34.3%), lack of time (32.1%), and pharmacist's advice (27%). Two-thirds of the self-medication users (68.6%) said that they already have antibiotics at home and the 43.2% said that they buy them directly at the pharmacy without a prescription.

### Participants' attitudes

The attitudes towards antibiotics use indicated that of the 67.3% respondents who had never be self-medicated with antibiotics, approximately one-quarters (22.7%) were of the opinion that they would be willing to take an antibiotic without a prescription of a physician. The analysis of the results of multivariate logistic regression for this outcome showed that respondents were more likely to be willing to be self-medicated if they had a lower self-rated health status (OR = 0.79; 95% CI = 0.65–0.95), if they did not know that antibiotics are not indicated for treating flu (OR = 0.42; 95% CI = 0.22–0.82) and sore throat (OR = 0.51; 95% CI = 0.27–0.95), and if they know that antibiotics are not indicated for treating cold (OR = 2.49; 95% CI = 1.04–5.95). Moreover, respondents under 40 years of age were more inclined to take an antibiotic without a prescription than those over 45 years (OR = 2.34; 95% CI = 1.22–4.5) (Model 3 in Table 2).

### Participants' major sources of knowledge

Questions concerning sources of information for the study population indicated that the majority acquired their antibiotics knowledge from physicians (80.1%); other sources in order were pharmacists (46.4%), newspapers/broadcast (18.8%), and internet (16.6%). About two-thirds (65.7%) of participants stated that more information regarding antibiotics were considered useful.

## Discussion

This study sought to identify the knowledge, attitudes, and behaviors of the Italian general population regarding antibiotics issues and to ascertain if there were factors associated with these main outcomes of interest.

The present study indicate some superficiality in knowledge about antibiotics that seemed particularly evident among the participants. For example, only 9.8% and 21.2% correctly knew the definition of antibiotic resistance and when it was appropriate to use antibiotics. Most of the respondents knew that antibiotics are not effective for cold, but erroneously believed that antibiotics can be used for sore throat, flu, and fever. In a large telephone survey among adults in Hong Kong, 91% had heard the term of antibiotic resistance [25], and in the general public in Malaysia respectively about 59% and 67.2% knew that overuse of antibiotics could cause antibiotic resistance and incorrectly thought that antibiotics are also used to treat viral infections [27]. A survey on adults in the United Kingdom showed that 38% did not know that antibiotics do not work on most coughs and colds [17]. In a survey on caregivers on the use of antibiotics for children in Mongolia, there was a lack of knowledge since many respondents gave incorrect answers about antibiotic use for cold or flu (83%), cough (81%), sore throat (74%) or purulent nasal discharge (64%) [28]. Furthermore, less than one-third of medical students in Congo correctly answered to install no antibiotic treatment in case of upper respiratory tract infection [29]. Direct comparison of the results observed in the present study with those in the literature is difficult. The findings may vary considerably among countries and this may partly be attributable to the use of different instruments, differences from one setting to another, selection of demographic and ethnically diverse study population, health status of the population, health-care delivery system, and health-care utilization.

The attitudes towards antibiotic use indicated that approximately one-quarter of the respondents were of the opinion that they would be willing to take an antibiotic without a prescription of a physician. This value was similar to the 18.7% of adults, both Jewish and Arab, in northern Israel who would consider self-medication with antibiotics without a medical consultation [30], whereas, in Turkey, the 54.1% of the participants stated their intention to use self-medication with antibiotics [23].

Regarding the use of self-medication with antibiotics, one-third (32.7%) of the respondents self-classified them as users of self-medication since they had taken an antibiotic without a prescription. This value was higher than the 23% of ever users reported in a survey in 11 European countries [10] and the 9% found for upper respiratory tract infections in the community of Hong Kong [2]. The prevalence of self-medication among tertiary level students in Ghana was 70% [22] and 44.1% of university students in Turkey started self-medication when they became ill [31]. The percentage of self-medication in the last 12 months was 19.1% among primary healthcare center attendants in Turkey [23], 46% by the community in the United Arab Emirates [32], and 53.2% in the general population of Lithuania [33]. Values respectively of 41% and 42.3% have been observed during 6 months before the query in Spain among Finnish immigrants [1] and in caregivers who had used non-prescribed antibiotics to treat symptoms in their child in Mongolia [28], and of 47.8% in Chinese university students [34]. Finally, 17.7% of the Spanish adult population who took antibiotics during the previous 2 weeks did so in the form of self-medication [35].

The multivariate analysis showed that, among the background characteristics of the study population, educational level, age, and working activity were found to be important indicators of the outcomes of interest. Lower educational level and unemployed status were identified as significantly associated for not knowing the definition of antibiotic resistance. The lack of knowledge among those with lower education has been already described in other studies conducted in several countries [2], [16]–[18], [27], [36], [37]. Among respondents who had never self-medicated with antibiotics, the young population was more inclined to take an antibiotic without a prescription of a physician than those older. Previous studies have documented the role of the younger age as significant predictors of intended self-medication [38]. The findings from the multivariate analysis indicated that it is necessary to reinforce antibiotics education among unemployed and less educated population.

As expected health-care providers, who represent the major source of acquiring information on antibiotics in the present survey, have been shown to significantly influence the use of self-medication since it was more frequently in those who did not use this source of information. However, it should be noted that there is a possibility of incongruities in expression of behavior: those who have attended a physician in the last year were more likely to use self-medication. These facts suggest once again that primary care physicians and other health-care providers are important and trustworthy components of any health education and promotion programs that seeks to increase appropriate behaviors. Thus, physicians, especially general practitioners, ought to strengthen health education and guidance and accurate information about antibiotics must be made available so that the general population can acquire knowledge especially regarding treatments and importance of the antibiotics. This study also pointed out the information needs on antibiotics indicated by almost two-thirds of the respondents. The importance of the information has been also revealed with the finding that those who have no such need had a higher knowledge level.

The study participants reported that sore throats was the most frequently cited reason for self-medication with antibiotics. This is in corroboration with a large body of previous similar surveys around the world [1], [21], [34], [38], [39]. Interestingly, other studies reported different diseases and symptoms as major reasons for self-treatment with antibiotics, such as tonsillitis [32], upper respiratory tract infections [40], common cold [22], [31], fever [25], [41], and cough [28], [42], [43].

Interpretation of the findings of this study should take into account certain potential limitations that might impact upon the conclusions drawn. Firstly, this study was based on a cross-sectional design, which precludes any precise conclusion regarding the causal relationships between the dependent and independent variables. Secondly, as with most surveys with self-administered questionnaire, there is the possibility that participants may over-report socially desirable behaviors or under-report socially undesirable behaviors. There were no mechanisms to objectively assess the honesty of the participants' answers to the survey questions. The absence of identifying data on the questionnaire sheets and confidentiality would tend to minimize such bias. Thirdly, the questionnaire has been filled out at home and it is possible that some respondents reading recommended materials had thus acquired a greater knowledge prior to questioning. As a result, the low level of knowledge found in this sample may be likely to represent a more favorable picture than would be observed in an average general population. Despite the limitations described above, these findings extended previous research and provide important information for evaluating and improving the understanding about antibiotics.

The present survey has generated information about knowledge, attitudes, and behaviors regarding antibiotics issues in the general population and effective public education initiative should not only disseminate information, but also provide practical and appropriate means to change their behavior.
